# Factors associated with severe respiratory syncytial virus infection among hospitalized children in Thammasat University Hospital

**DOI:** 10.12688/f1000research.146540.1

**Published:** 2024-03-27

**Authors:** Pornumpa Bunjoungmanee, Samita Sompoch, Auchara Tangsathapornpong, Prapasri Kulalert

**Affiliations:** 1 Department of Pediatrics, Faculty of Medicine, Thammasat University, Amphoe Khlong Luang, Pathum Thani, 12120, Thailand; 2 Department of Clinical Epidemiology, Faculty of Medicine, Thammasat University, Khlong Luang District, Pathum Thani, 12120, Thailand

**Keywords:** respiratory syncytial virus (RSV), lower respiratory tract infection (LRTI), severe RSV-LRTI

## Abstract

**Background:**

Respiratory syncytial virus (RSV) is one of the most significant respiratory pathogens that causes acute lower respiratory tract infections (LRTI) early in life. Most children have a history of RSV infection within 24 months of age, and recurrent infections are common throughout life.

**Methods:**

Children under five years of age were identified through a review of medical records with a diagnosis of RSV-LRTI between 2016 and 2020. Severe RSV-LRTI was defined as a prolonged length of stay (> 7 days), admission to the intensive care unit, need for mechanical ventilation, non-invasive positive pressure ventilation, or in-hospital mortality. Factors associated with severe RSV-LRTIs were investigated using univariate and multivariate analyses.

**Results:**

During the study period, 620 patients were diagnosed with RSV-LRTI and 240 (40.16%) patients had severe RSV-LRTI. In the multivariable logistic regression analysis, the factors for severe RSV-LRTI were being under 3 months (aOR 2.18 CI 1.39-3.43, p0.001), cardiovascular disease (aOR 3.55 CI 1.56-8.06, p0.002), gastrointestinal disease (aOR 5.91 CI 1.90-18.46, p0.002), genetic disease (aOR 7.33 CI 1.43-37.54, p0.017), and pulmonary disease (aOR 9.50, CI 4.56-19.80, p<0.001). Additionally, the presence of ≥ 2 co-morbidities (aOR 6.23 CI 2.81-14.81, p<0.016), experiencing illness for more than 5 days (aOR 3.33 CI 2.19-5.06, p<0.001), co-detection of influenza (aOR 8.62 CI 1.49-38.21, p0.015), and nosocomial RSV infection (aOR 9.13 CI 1.98-41.30, p0.012), markedly increased the risk of severe RSV-LTRI. The severe RSV-LRTI group demonstrated higher hospitalization expenses (median, US $720.77 vs $278.00, respectively; p<0.001), and three infants died in-hospital.

**Conclusion:**

Children at high risk for RSV-LRTI due to underlying genetic and gastrointestinal diseases are at an increased risk for severe RSV-LRTI. Further studies to determine the cost-effectiveness of RSV immunization in these potential co-morbidities should be initiated to prioritize RSV immunization, especially in resource-constrained regions with limited availability of nirsevimab.

## Introduction

Respiratory syncytial virus (RSV) is a viral pathogen with far-reaching consequences in children, and is associated with significant morbidity and mortality. Infants have an increased risk of developing severe RSV infection, often necessitating hospitalization. Hospitalized RSV-associated lower respiratory tract infections (RSV-LRTIs) occur globally. In Thailand, the highest peak incidence occurred during the rainy season from July to October. Those aged < 5 years experienced a higher mortality rate due to RSV-LRTI than older children, especially those aged < 1 year. ^1^

Many recent studies have demonstrated that young age, preterm birth, and pre-existing diseases are significant risk factors for RSV hospitalization. Interestingly, most children hospitalized for RSV are healthy. ^2^^–^^4^ The RSV season occurs annually in Thailand. ^1^ However, there are limited available data regarding hospitalization, utilization of medical resources, and risk factors for severe RSV-LRTIs. This retrospective study aimed to identify the clinical features and manifestations in hospitalized children with RSV-LRTI, along with the risk factors for severe RSV-LRTI and death. Demographic characteristics and disease severity were considered potential factors influencing the cost of medical treatment and utilization of medical resources.

## Methods

### Ethic statement

The Human Research Ethics Committee of Thammasat University (Medicine) is in full compliance with international guidelines such as Declaration of Helsinki, The Belmont Report, CIOMS Guidelines and the International Conference on Harmonisation-Good Clinical Practice (ICH-GCP), approved our study. The approval number is MTU-EC-PE-0-114/64 and the date of approval is May 20, 2021. Data collection was initiated after requisite approvals were obtained from the Human Research Ethics Committee of Thammasat University (Medicine).

This study received a waiver of informed consent due to its retrospective nature and the absence of direct contact with the study subjects. This study did not involve any intervention or therapy, thereby posing no risks to the subjects. Confidentiality of the present study data was maintained in accordance with the Declaration of Helsinki.

### Study design

This retrospective cross-sectional study was initiated at the Thammasat University Hospital (TUH) in Pathum Thani, Thailand, a tertiary care facility with 100 pediatric beds. This study was based on a systematic computer-assisted database search that allowed extraction of retrospective data of the patients aged < 5 years who were discharged with a diagnosis of RSV-LRTI, including clinical bronchitis, bronchiolitis, and pneumonia. Confirmation of the diagnosis was established either by an RSV antigen immunochromatography assay or a polymerase chain reaction (PCR) test for RSV from specimens taken from nasal or nasopharyngeal swabs.

The severe RSV-LRTI group included children who experienced an unsatisfactory outcome or died. An unsatisfactory outcome was defined as the necessity for non-invasive positive pressure ventilation (NIPPV), or mechanical ventilation, or prolonged hospital stay (over 7 days), in-hospital mortality, or admission to pediatric intensive care unit (PICU). The non-severe group included children with RSV-LRTI who did not experience an unsatisfactory outcome or death.

### Data collection

The study population was identified by reviewing inpatient medical records, including patients age 0-5 years old from 2016 to 2020. Factors associated with severe hospitalized RSV-LRTI included baseline characteristics, clinical manifestations, and initial laboratory findings. The baseline characteristics included demographic data and co-morbidities. Clinical manifestations included presenting symptoms, physical examination, and initial laboratory results consisting of electrolytes and complete blood counts. Hospital resource utilization, hospital cost data, and outcomes after hospital stay were collected for both groups. Data on the mode of oxygen supplementation, inotropic drug use, bronchodilator nebulizer, use of montelukast, antibiotic therapy, and blood transfusion were collected to assess hospital resource utilization. The cost data were sourced from the hospital’s cost-accounting database. An exchange rate of 35 baht per 1 US dollar was used to convert all expenditures in Thai baht into US dollars. The outcome after the hospital stay was recovery or in-hospital death. The term “nosocomial RSV-LTRI” was defined as signs or symptoms of RSV-LRTI occurring more than 72 h after admission.

The definitions of the variables included cyanotic heart disease or congenital hemodynamic significance, and heart disease was regarded as congenital heart disease (CHD). Cerebral palsy and other central nervous system abnormalities were defined as neurological diseases. Children born before 37 weeks of age were classified as preterm infants. Bronchopulmonary dysplasia (BPD) or asthma is a pulmonary disease. Necrotizing enterocolitis (NEC) with short bowel syndrome, intestinal malformation, esophageal atresia, or biliary atresia was defined as gastrointestinal disease. Hematological diseases included thalassemia and red cell membrane defects. Genetic diseases include Down syndrome, DiGeorge syndrome, Williams syndrome, and Rubinstein-Taybi syndrome.

### Data analysis

This retrospective study aimed to identify the factors associated with severe RSV-LRTI, including perinatal history, co-morbidities, clinical manifestations, and laboratory results. In addition, the assessment of hospital resource utilization for RSV-LRTI included the mode of oxygen supplementation, inotropic drug use, bronchodilator nebulizer, use of montelukast, antibiotic therapy, and blood transfusion.

Categorical data were expressed as frequencies and percentages. Continuous data are expressed as medians with interquartile ranges (IQRs). Fisher’s exact test was used to compare categorical data. The Wilcoxon rank-sum test was used to compare continuous data. Univariate and multivariate analyses were conducted to ascertain the independent factors associated with severe RSV-LRTI (p<0.05).

Frequencies and percentages were used for categorical data. The median and interquartile range (IQRs) were used for continuous data. Fisher’s exact test and Wilcoxon rank-sum test were used to compare the categorical and continuous data, respectively. Univariate and multivariate analyses were conducted to ascertain the independent factors associated with severe RSV-LRTI (p<0.05).

## Results

**Figure 1.**
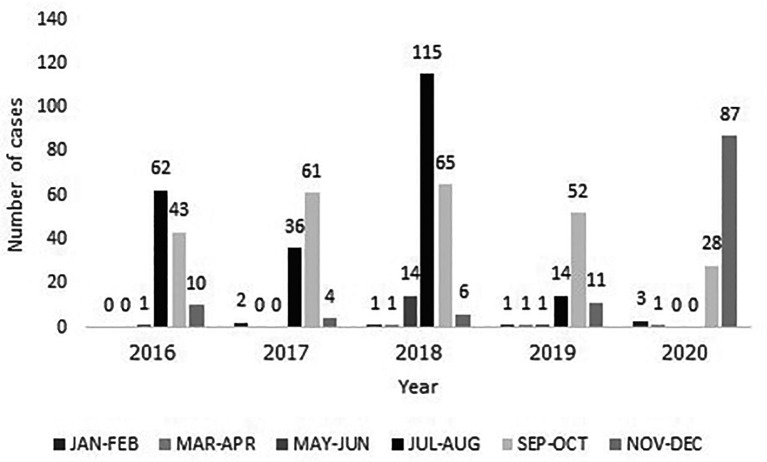
The distribution of RSV-LRTI children, 2016-2020.

Overall, 1,050 children were admitted for a positive RSV test result. In this study, 620 children diagnosed with RSV-LRTI were included; 10 cases were excluded because of missing values. Baseline characteristics of the patients are shown in Table 1. Of the 620 patients, 249 had severe RSV-LTRI. The mean age of all patients was 16.60 ± 14.56 months old and males accounted for 53.55 percent of all patients. One-third of patients had at least one comorbidity. Eighteen patients (2.74%) had nosocomial RSV infection, and 11 patients (1.61%) had co-infection with influenza, which occurred specifically in the severe group. The peak of the RSV-LRTI was noted from July to October annually, as demonstrated by the seasonal variation in RSV ( Figure 1). Most patients were admitted to the general pediatric ward (96.77%), with an average length of stay of 5.87 ± 2.43 days. A total of 3.23% of patients required PICU admission ( Table 2).

**Table 1.** Baseline characteristics of children hospitalized for RSV-LRTI, 2016-2020.

Characteristics	Sever RSV-LRTI N = 249	Non-severe RSV-LRTI N = 371	Total N = 620	p-value
Age, months, mean ± SD	15.45 ± 14.76	17.37 ± 14.39	16.60 ± 14.56	0.017
Age range, n (%)				
0-3 mo	62 (24.90)	63 (17.00)	125 (20.16)	0.019
>3-6 mo	26 (10.44)	42 (11.31)	68 (10.97)	
>6-12 mo	53 (21.29)	69 (18.60)	122 (19.68)	
>12-24 mo	48 (19.27)	98 (26.41)	146 (23.55)	
>24 mo	60 (24.10)	99 (26.68)	159 (25.64)	
Male, n (%)	131 (52.61)	201 (54.18)	332 (53.55)	0.743
Co-morbidities, n (%)				
Preterm	34 (13.65)	16 (4.31)	50 (8.06)	<0.001
Pulmonary disease	51 (20.48)	11 (2.96)	62 (10.00)	<0.001
Gastrointestinal disease	30 (12.05)	4 (1.08)	34 (5.48)	<0.001
Congenital heart disease	27 (10.84)	12 (3.23)	39 (6.29)	<0.001
Hematologic disease	12 (4.82)	6 (1.62)	18 (2.90)	0.027
Genetic disease	11 (4.42)	2 (0.54)	13 (2.10)	0.001
Neuromuscular disease	4 (1.61)	3 (0.81)	7 (1.23)	0.680
≥2 co-morbidities	43 (17.27)	10 (2.70)	52 (8.55)	<0.001
Duration of illness (days), mean ± SD	4.23 ± 1.34	3.52 ± 1.41	3.80 ± 1.42	<0.001
Onset > 3 days, n (%)	224 (89.96)	282 (76.01)	506 (81.61)	<0.001
Onset > 5 days, n (%)	111 (44.58)	65 (17.52)	176 (28.39)	<0.001
Co-infection with influenza, n (%)	10 (4.02)	1 (0.27)	11 (1.77)	0.001
Nosocomial RSV infection, n (%)	17 (6.83)	1 (0.27)	18 (2.90)	0.001
Symptoms, n (%)				
Cough	246 (98.80)	371 (100)	617 (99.52)	0.064
Rhinitis	241 (96.79)	353 (95.15)	594 (95.81)	0.415
Tachypnea	220 (88.35)	254 (68.46)	474 (76.45)	<0.001
Nausea/vomiting	84 (33.73)	125 (33.69)	209 (33.71)	1.000
Diarrhea	52 (20.88)	55 (14.82)	107 (17.26)	0.052
Poor intake	134 (53.82)	175 (47.17)	309 (49.84)	0.120
Sore throat	4 (1.61)	4 (1.08)	8 (1.29)	0.720
Cyanosis	6 (2.41)	0 (0.00)	6 (0.97)	0.004
Apnea	15 (6.02)	3 (0.81)	18 (2.90)	<0.001

Abbreviations: SD, standard deviation; RSV-LRTI, respiratory syncytial virus associated with lower respiratory tract infections.

**Table 2.** Initial clinical manifestation and outcomes of children hospitalized for RSV-LRTI, 2016-2020.

Characteristics	Sever RSV-LRTI N = 249	Non-severe RSV-LRTI N = 371	Total N = 620	p-value
Fever, °C, mean ± SD	38.08 ± 0.88	37.77 ± 0.81	37.89 ± 0.85	<0.001
Fever > 38.5°C, n (%)	69 (27.71)	61 (16.44)	130 (20.97)	0.001
Saturation (SatO _2_), %, mean ± SD	93.08 ± 4.43	96.07 ± 2.79	94.87 ± 3.83	<0.001
Desaturation (SatO _2_ < 95%)	144 (57.83)	66 (17.79)	210 (33.87)	<0.001
Dyspnea	209 (83.94)	242 (65.23)	451 (72.74)	<0.001
Chest retraction	234 (93.98)	284 (76.55)	518 (83.55)	<0.001
Fine crepitation	208 (83.53)	256 (69.0)	464 (74.84)	< 0.001
Rhonchi	136 (54.62)	189 (50.94)	325 (54.42)	0.412
Wheezing	107 (42.97)	94 (25.34)	201 (32.42)	<0.001
Initial PICU admission, n (%)	20 (8.03)	0 (0.00)	20 (3.22)	<0.001
Transmitted to PICU, n (%)	25 (10.04)	0 (0.00)	20 (4.03)	<0.001
Length of stay, day, mean ± SD	11.28 ± 14.10	3.48 ± 1.20	5.87 ± 2.43	<0.001
Deaths, n (%)	3 (1.20)	0 (0.00)	3 (0.48)	0.064
Cost (US $), median (P25 ^th^ -75 ^th^)	720.77 (503.77-1107.57)	278.00 (200.86-386.29)	387 (245.99-652.77)	<0.001

Abbreviations: SD, standard deviation; PICU, pediatric intensive care unit; RSV-LRTI, respiratory syncytial virus-associated lower respiratory tract infection.

The medical data of patients with severe and non-severe RSV-LRTIs at admission were compared ( Table 2). Most of the patients had cough, rhinitis, shortness of breath, and feeding difficulties (99.52%, 95.81%, 76.45% and 49.84%, respectively). However, the symptoms were not different in both groups, except apnea (p<0.01), tachypnea (p<0.001), and cyanosis (p0.04). At initial presentation, 130 patients (20.97%) had fever > 38.5 °C and 210 patients (33.87%) had desaturation, especially in the severe RSV-LTRI group. At first presentation, wheezing was documented in 201 patients (32.42%), comprising 107 patients with severe RSV-LRTI and 94 with non-severe RSV-LRTI ( Table 2). There were no differences in the complete blood count or serum electrolyte levels between the groups.

Healthcare utilization for RSV disease included hospital and PICU admissions, as well as treatments such as oxygen therapy, mechanical ventilation, inhaled medications, and antibiotics for managing RSV infection. The severe RSV-LTRI group showed significantly increased healthcare utilization and costs, especially in PICU admissions, and increased length of stay.

Oxygen therapy was prescribed to 504 (81.29%) patients. One-third of patients needed supplementary high-flow or positive-pressure oxygen administration, which included heated humidified high-flow nasal cannula (HHHFNC) (25.64%), nasal continuous positive airway pressure therapy (NCPAP) (5.49%), and invasive mechanical ventilation (IMV) (4.03%), exclusively observed in the severe RSV-LRTI group. The duration of oxygen therapy tended to be more prolonged in severe RSV-LTRI group compared to non-severe RSV-LRTI group (7.71 + 6.96 days vs. 2.35 + 0.96 days, p< 0.001) ( Table 3).

**Table 3.** Therapy of children hospitalized for RSV-LRTI, 2016-2020.

Therapy	Sever RSV-LRTI N = 249	Non-severe RSV-LRTI N = 371	Total N = 620	p-value
Respiratory support, n (%)	243 (97.59)	261 (70.35)	504 (81.29)	<0.0001
Oxygen canula	222 (89.16)	264 (71.16)	486 (78.39)	<0.001
NCPAP/HHHFNC	193 (77.51)	0 (0.00)	193 (31.13)	<0.001
Mechanical ventilator	25 (10.04)	0 (0.00)	25 (4.03)	<0.001
Duration of oxygen support, day, mean ± SD	7.71 ± 6.96	2.35 ± 0.96	4.93 ± 5.57	<0.001
Inhaled salbutamol, n (%)	208 (83.53)	222 (59.84)	430 (69.35)	<0.001
Duration of administration for inhaled salbutamol, day, mean ± SD	3.68 ± 3.52	1.78 ± 1.07	2.70 ± 2.73	<0.001
Inhaled hypertonic saline, n (%)	137 (55.02)	80 (21.56)	217 (35.00)	<0.001
Duration of administration for inhaled hypertonic saline, day, mean ± SD	5.01 ± 3.49	2.29 ± 0.93	4.01 ± 3.12	<0.001
Inhaled adrenaline, n (%)	169 (67.87)	142 (38.27)	311 (50.16)	<0.001
Duration of administration for inhaled adrenaline, day, mean ± SD	4.04 ± 2.24	2.17 ± 1.12	3.19 ± 2.04	< 0.001
Montelukast, n (%)	63 (25.30)	34 (9.16)	97 (15.65)	0.412
Blood transfusion	11 (4.42)	0 (0.00)	11 (1.77)	<0.001
Receiving any antibiotic treatment, n (%)	147 (59.04)	26 (7.01)	173 (27.90)	<0.001
Cefotaxime/ceftriaxone	114 (45.78)	16 (4.31)	130 (20.96)	
Azithromycin	19 (7.63)	2 (0.54)	21 (3.39)	
Meropenem	17 (6.83)	0 (0.00)	17 (2.74)	
Ceftazidime/cefoperazone-salbactam	8 (3.21)	0 (0.00)	8 (1.29)	
Ampicillin	7 (2.81)	0 (0.00)	7 (1.23)	
Duration of antibiotic treatment, day, mean ± SD	7.66 ± 2.20	6.08 ± 1.69	7.42 ± 2.20	<0.001
Oseltamivir, n (%)	25 (10.04)	1 (0.27)	26 (4.19)	<0.001
Systemic steroid, n (%)	35 (14.06)	9 (2.43)	44 (7.10)	<0.001

Abbreviations: SD, standard deviation; RSV-LRTI, respiratory syncytial virus-associated lower respiratory tract infection; NCPAP, nasal continuous positive airway pressure; HHHFNC, heated humidified high-flow nasal cannula.

In cases of severe RSV-LRTI, the prescription rates and the duration usage for salbutamol, adrenaline, and hypertonic saline nebulization were notably higher compared to the non-severe RSV-LRTI (83.53% vs. 59.84% and 3.68 ± 3.52 days vs. 1.78 ± 1.07; p< 0.001, 67.87% vs 38.27% and 4.04 ± 2.24 days vs. 2.17 ± 1.12 days; p< 0.001, 55.02% vs. 21.56% and 5.01 ± 3.49 days vs. 2.29 ± 0.93; p<0.001, respectively). A total of 173 patients (27.90%) received treatment with antibiotics; ceftriaxone/cefotaxime was the most common antibiotic used in both groups (34.94% in severe RSV-LRTI and 4.31% in non-severe RSV-LRTI) ( Table 3). The severe RSV-LRTI group had prolonged length of hospital stay (PLOS) (mean, 11.28 ± 14.10 days vs. 3.48 ± 1.20 days, p<0.001), higher mortality (1.20% vs. 0%; p0.064), and excess total hospital costs (median, US $720.77 vs $278, p0.001). There were 29 cases in the severe RSV-LRTI group were transmitted from the general pediatric ward to the PICU ( Table 3).

Three infants died in the hospital, with a mortality rate of 0.48%. All mortality cases had co-morbidities. A cardiac anatomical defect was observed in two cases (one case had Tetralogy of Fallot, and the other case had atrial septal defect with ventricular septal defect). Furthermore, Down syndrome with gastroesophageal reflux disease and subglottic stenosis was observed in one patient. All three cases were complicated by nosocomial bacterial infections, namely *Klebsiella pneumonia* and *Acinetobacter baumanii,* which led to a lethal outcome.

In univariate analysis, the risk factors associated with severe RSV-LRTI were being under 3 months (aOR 1.62, CI 1.09-2.40, p0.017), hematologic disease (aOR 3.08 CI 1.14-8.32, p0.026), preterm birth (aOR 3.51 CI 1.89-6.51, p<0.001), cardiovascular disease (aOR 3.64 CI 1.81-7.33, p<0.001), pulmonary disease (aOR 8.43 CI 4.30-16.54, p<0.001), genetic disease (aOR 8.53 CI 1.87-38.81, p0.006), and gastrointestinal disease (aOR 12.57 CI 4.37-36.15, p<0.001). Neurological diseases and immunocompromised status did not differ between the groups. Furthermore, the presence of ≥ 2 co-morbidities significantly increased the risk of severe RSV-LTRI (aOR 7.53 CI 3.70-15.32, p<0.001). The duration of illness was associated with a severe RSV-LRTI. Experiencing illness for more than three days (aOR 2.83 CI 1.75-4.56, p<0.001), particularly exceeding five days (aOR 3.78 CI 2.62-5.46, p<0.001), was associated with severe RSV-LRTI. Moreover, co-detection of influenza (aOR 9.78 CI 1.96-40.12, p0.005) and nosocomial RSV infection (aOR 10.65 CI 2.14-43.42, p0.004), increased the risk of severe RSV-LRTI.

Multivariate analysis showed that <3 months (aOR 2.18 CI 1.39-3.43, p0.001), cardiovascular disease (aOR 3.55 CI 1.56-8.06, p0.002), gastrointestinal disease (aOR 5.91 CI 1.90-18.46, p0.002), genetic disease (aOR 7.33 CI 1.43-37.54, p0.017), and pulmonary disease (aOR 9.50 CI 4.56-19.80, p<0.001) were predictive factors for severe RSV-LRTI. Moreover, the presence of ≥ 2 co-morbidities markedly increased the risk of severe RSV-LTRI (aOR 6.23 CI 2.81-14.81, p<0.016). Regardless of co-morbidities, experiencing illness for more than 5 days (aOR 3.33 CI 2.19-5.06, p<0.001), co-detection of influenza (aOR 8.62, CI 1.49-38.21, p0.015), and nosocomial RSV infection (aOR 9.13 CI 1.98-41.30, p0.012) were associated with a higher risk of severe RSV LRTI ( Table 4).

**Table 4.** Univariable and multivariable analysis of risk factor for severe RSV-LRTI, 2016-2020.

Characteristics	Univariable logistic regression OR (95% CI), p-value	Multivariable logistic regression aOR (95% CI), p-value
Age under 3 months	1.62 (1.09-2.40), 0.017	2.18 (1.39-3.43), 0.001
Gastrointestinal disease	12.57 (4.37-36.15), <0.001	5.91 (1.90-18.46), 0.002
Genetic disease	8.53 (1.87-38.81), 0.006	7.33 (1.43-37.54), 0.017
Pulmonary disease	8.43 (4.30-16.54), <0.001	9.50 (4.56-19.80), <0.001
Congenital heart disease	3.64 (1.81-7.33), <0.001	3.55 (1.56-8.06), 0.002
Preterm	3.51 (1.89-6.51), <0.001	1.66 (0.80-3.44), 0.170
Hematologic disease	3.08 (1.14-8.32), 0.026	2.88 (0.95-8.70), 0.061
≥2 co-morbidities	7.54 (3.70-15.31), <0.001	6.23 (2.81-14.81), 0.016
Co-infection with influenza	9.78 (1.96-40.12), 0.005	8.62 (1.98-41.30), 0.012
Nosocomial RSV infection	10.65 (2.14-43.42), 0.004	9.13 (1.98-41.30), 0.012
Onset of illness > 3 days	2.83 (1.75-4.56), <0.001	1.74 (1.43-37.54), 0.047
Onset of illness > 5 days	3.78 (2.62-5.46), <0.001	3.33 (2.19-5.06), <0.001

Abbreviations: RSV-LRTI, respiratory syncytial virus associated lower respiratory tract infection; OR, odds ratio; aOR, adjusted odds ratio; 95% CI, 95% confidence interval.

## Discussion

Evaluating the data collected over five consecutive years, we found that 40 percent of the children demonstrated severe symptoms. Notably, the predominant proportion of severe RSV-LRTI cases was observed in infants, particularly in those aged < 3 months. Gastrointestinal diseases, such as NEC with short bowel syndrome, intestinal malformation, or esophageal atresia, were additional significant risk factors for severe RSV-LRTI. Gut microbiome dysbiosis in gastrointestinal anomalies may play a key role in RSV infections. The role of the gut microbiota in regulating the immune system and respiratory infections is increasingly recognized. ^5^ A relationship between gut microbiome dysbiosis and RSV infection was demonstrated in a previous study. Disruptions in microbial abundance and characteristic microbiome shifts are associated with RSV severity. ^6^

Down syndrome, with or without congenital heart defects, has a higher risk of mortality, prolonged length of hospital stay, and more frequent transfer to the PICU. ^7^^,^^8^ Our study observed a correlation between genetic disease and RSV-LRTI with 80% (9/11) of genetic diseases in the severe RSV-LRTI group with Down syndrome, which demonstrated a 7-fold higher risk for severe RSV-LRTI. Therefore, gastrointestinal anomalies, short bowel syndrome, and Down syndrome should be considered as candidates for RSV immunization.

Other factors related to severe RSV-LRTI were co-morbidities of cardiovascular disease, pulmonary disease, and co-detection of influenza, which is comparable to the findings of previous studies. ^4^^,^^7^^,^^9^^–^^12^ Many studies have indicated that prematurity is a risk factor for severe RSV-LRTI; however, this was not statistically significant in this study. Moreover, the cumulative number of co-morbidities is a potential factor associated with a severe course of hospitalization. ^7^ Children with two or more co-morbidities were at a significantly higher risk of severe RSV-LRTI. Nevertheless, one-fourth of patients with co-morbidities were ineligible for RSV immunization. Further studies are required to determine the cost-effectiveness of immune prophylaxis against RSV for other potential co-morbidities.

Nosocomial RSV infection is identified as one of the factors associated with mortality and PICU admission. ^13^^,^^14^ Previous reports have suggested that nosocomial RSV infection is an independent predictor of prolonged hospitalization, higher mortality, and excess hospital charges. ^15^ Our results showed that nosocomial RSV infection was significantly associated with severe RSV-LRTIs. Nonetheless, the small number of nosocomial RSV cases limited our ability to detect a correlation between nosocomial RSV infection and mortality.

The duration of illness prior to admission for more than three days, especially exceeding five days, was considered a risk factor for severe RSV-LRTI, as in a previous study. ^16^ This could be explained by the prolonged duration of illness, which may be associated with complications, notably, atelectasis. Following RSV infection, there is an increase in the quantity and viscosity of the mucous secretions. The loss of ciliated epithelial cells creates widespread mucous plugging across various areas. ^17^ Furthermore, secondary bacterial infections may play a key role in the severity of symptoms, particularly in infants. RSV infection diminishes bacterial clearance, leading to secondary bacterial pneumonia by altering the recruited neutrophils. ^18^^,^^19^ Nevertheless, our study did not demonstrate a statistically significant association between secondary bacterial infection and severe RSV-LRTI. This was due to the fact that requests for sputum culture and blood culture in RSV-LRTI are optional and depend on the judgment of the attending physician.

The RSV seasons vary globally and are influenced by climate and geographic location. Several studies have demonstrated a relationship between RSV activity and weather conditions. ^20^^,^^21^ Our 2016-2020 study established a correlation between the rainy season (July–October) and RSV-LRTI admission. This seasonal pattern was similar to that reported in previous studies in Thailand and other Southeast Asian countries. ^1^^,^^9^^,^^22^^–^^24^ However, RSV-LRTI admissions in 2020 predominantly occurred between September and December because of the delayed onset of the rainy season. Furthermore, communities and academic institutions reopened after the relaxation of COVID-19 lockdown measures starting in August 2020, resulting in an upsurge in RSV-LRTI admissions, notably from September.

Management of children hospitalized with RSV infection involves supportive care and should include hydration and if necessary supplemental oxygen, if necessary. In our study, bronchodilators, epinephrine, montelukast, and corticosteroids were used in the treatment of severe RSV LRTI with statistical significance. However, there is no clinical data to recommend these medications for the treatment of RSV-LRTIs. ^25^ Likewise, in previous studies, ^9^^,^^26^ 27.9% of our patients were prescribed antibiotics, demonstrating a notably higher frequency of severe RSV-LRTIs (59.04% vs. 7.01%). Although antibiotics were prescribed to treat a possible bacterial superinfection in severe RSV-LRTI, ^19^ the misuse and overuse of antibiotics for RSV infection was established in a previous study. ^27^ Overprescription of antibiotics is the main cause of adverse consequences, not only adverse reactions from the antibiotics but also unnecessary economic burden, financial stress, ^28^ or antibiotic resistance. ^29^ This result emphasizes the necessity of implementing appropriate antibiotic stewardship programs that have demonstrated effectiveness in reducing antibiotic misuse in RSV-infected children. ^30^^,^^31^

Therefore, prevention of RSV infection is necessary. Nirsevimab is a new monoclonal antibody that adheres to the perfusion protein on the surface of RSV and has a longer half-life than that of palivizumab. Since October 2023, the United States Centers for Disease Control and Prevention (U.S. CDC) has recommended RSV immunoprophylaxis with nirsevimab for all infants younger than 8 months who are born during the 2023-2024 RSV season and should be used in place of palivizumab. However, both nirsevimab and palivizumab are not available in Thailand. Currently, there is no approved vaccine for RSV infection in children. Many RSV vaccines targeting infants and young children are undergoing development and employ diverse technologies such as protein subunits, live-attenuated viruses, messenger RNA, or viral vector techniques to stimulate immune responses. ^32^^,^^33^

Maternal immunization is another strategy for preventing RSV infections in infants. Since August 2023, the U.S. Centers for Disease Control and Prevention (CDC) has recommended a single intramuscular injection of inactivated non-adjuvanted recombinant RSV vaccine during weeks 32 through 36 of pregnancy. The goal of vaccination is to provide passive protection against RSV in infants during the first few months of life. However, RSV vaccines for pregnancy are not available in Thailand.

This study had some limitations. Despite the relatively high number of patients, the study had a retrospective design and was conducted at a single medical center, which might potentially limit the generalizability of the results. Additionally, the analysis of co-infection was limited because an RT-PCR assay for respiratory viral panels was not feasible in all cases. Evidence of secondary bacterial infection was unavailable in most patients due to the lack of sputum and blood cultures performed in most patients. Furthermore, the study was conducted in a tertiary academic medical center, which may have a population selection bias, including children with chronic conditions.

## Conclusion

RSV infection is a major cause of respiratory hospitalization in children. Mortality and morbidity occur frequently in younger infants. Co-morbidities, including gastrointestinal anomaly, short bowel syndrome, Down syndrome, and cardiopulmonary function, are significantly higher risk factors for severe RSV-LRTI. Moreover, the disease severity of RSV-LRTI is correlated with being under 3 months of age, co-infection with influenza, nosocomial RSV infection, and prolonged duration of illness. The primary treatment for RSV infection is supportive care. There are no specific antiviral therapies or vaccines for RSV in children. Effective preventive measures with RSV immune prophylaxis should be prioritized in public health policies and primarily target all infants and children with risk factors to provide coverage throughout the RSV season.

## Data Availability

Factor associated with severe respiratory syncytial virus infection among hospitalized children in Thammasat University Hospital. The anonymized data sets of this project are available in the Zenodo: https://doi.org/10.5281/zenodo.10408423. ^34^ Data are available under the terms of the Creative Commons Attribution 4.0 International license (CC-BY 4.0).
