# Supplementary material for: Factors associated with severe respiratory syncytial virus infection among hospitalized children in Thammasat University Hospital
Source: F1000Res. 2024 May 17;13:231. Originally published 2024 Mar 27. [Version 2] doi: 10.12688/f1000research.146540.2 (PMC11269972; doi:10.12688/f1000research.146540.2)
Supplement: Peer review history

# Reviewer report: Reviewer response for version 2

**DOI:** 10.5256/f1000research.165923.r298255

**Published:** 2024-07-24

**Authors:** Heloise Buys

**Affiliations:** 1 Ambulatory & Emergency Paediatrics, Red Cross War Memorial Children's Hospital, University of Cape Town Department of Paediatrics and Child Health (Ringgold ID: 63732), Rondebosch, Western Cape, South Africa

**REPORT**

 Thank you for the opportunity to review “ Factors associated with severe respiratory syncytial virus infection among hospitalized children in Thammasat University Hospital [version 2; peer review: 1 approved]” by Bujoungmanee et al.

 This is a retrospective observational study, carried out at a tertiary academic hospital in Thailand over 2016-2020  conducted in an upper-middle-income-setting, that attempts to understand the challenges and associated factors involved with severity of RSV-associated respiratory illness in children aged <5years. The study was carried out via medical records review. The discussion is quite lengthy.

 The researchers have completed a fairly comprehensive and well written  investigation and have managed to cast light on which children may be more vulnerable to severe RSV-associated LRTI- they suggest that this may identify these as being most likely to benefit from targeted protective interventions.

**Compliance with journal-specific instructions**

 Mostly yes- will indicate in the report specific areas where there is deviation.

**Title**

 Clear, though strictly should indicate that this is a retrospective cross-sectional descriptive study.

**Abstract**

 Provides a good summary of the main findings, although it lacks the aim or purpose of the study.

 The keywords are adequate.

**Methodology**

 We are told that the 620 patients were recruited from a total of 1 050 children with RSV positive upper respiratory swabs; it is not clear why 420 children were excluded ( a simple flow diagram may clear this). The data collection is well-described- a sentence of explanation will help the reader decide on context and generalisability. The exclusion/inclusion criteria of the sample population are not explicitly included.

- The study period is defined but the sample size determination is not fully described.

 Data collected included n, age, clinical characteristics and co-morbidity  data , timing-related factors, clinical and operational data. This information was tabulated. I believe that your text notes about the seasonality of RSV-infection is adequate without the need for Figure 1.- just a suggestion.

 The statistical analysis section is covered reasonably.

**Results**

 The first sentence in the results section requires some minor editing to indicate the real reason for admission.

 The results section has four tables which contain enough information for the reader to interpret without the lengthy repetition in the text paragraphs. Please consider reducing the text.

 Table 1: summarises the baseline characteristics very well. I wonder somewhat at two of the variables- sore throat- is difficult to understand given that >70% of the study children would not have been able to provide this information due to their young age, making this variable less useful for inclusion. Likewise, cyanosis is quite a subjective observation with much inaccuracy and inter-observer variability- most of us cannot detect this until significant desaturation has occurred- therefore, why not use the World Health Organization cut-off for hypoxia- <92%?

 This goes for Table 2 as well, where desaturation is given at an arbitrary <95%- it would help to understand how this level of desaturation was decided upon.

  Table 2 is a good table where the severity indicators are depicted and compared, albeit in univariable analysis. The first row of the table is likely redundant seeing as fever is explored more meaningfully in the second row.

 Table 3 describes the various treatments used on the study children, the most important of which regard the respiratory support required by the children- likely the most appropriate indicators of severity of illness.

 Given the current body of evidence and guidelines such as NICE and WHO with respect to the management of bronchiolitis, finding significance in the use of the other treatments listed in those who had severe disease compared to those with non-severe disease is simply a description of measures used in the sicker children at a single tertiary centre. There is no evidence given that the effects of these treatments were evaluated  as beneficial in detail in this retrospective observational study.

**Reproducibility**

 Yes

**Major concerns**

 Nil

**Minor concerns**

 Paragraphs in the Results section which describe what is depicted n Table 4 are unnecessary as    Table 4 illustrates the findings very well.

 Ensure consistency of terminology: univariable and multivariable regressions are correctly used, compared to univariate  and multivariate terms used in the data analysis section of the methods

**Reference section**

 I would question whether reference 40 is a true reference, even though it refers to a pre-print, it is unconventional to self-reference one’s own current paper.

**Discussion**

- The discussion is very long and I would suggest that it should open with a bold appropriate summary of the main findings of the study.
- The main findings are described, and the  relevant published literature is drawn in to compare and contrast cogently. I would suggest that not all findings should be addressed- choose the most impactful.
- The association of GIT diseases with disease severity made me speculate on the role played by of nutritional status’ on these affected  children- this was not mentioned in their baseline characteristics- do we have any information on this, i.e., were any of the study cohort malnourished in any way?
- I would suggest that the tertiary centre’s management of children with bronchiolitis with less conventional therapies other those recommended by NICE and WHO Guidelines, has its greatest impact on hospital resources, perhaps this consideration could reduce the paragraph length.
- The impact of prevention by vaccination is mentioned appropriately but cannot be added to the conclusions as the study did not specifically address this.

**Conclusions**

 The conclusion section is much too long and rehashes some of the background and results, it should be quite clear and concise, one or two sentences and with a sentence on possible future research. I would suggest also shortening the recommendations section to the most important points, e.g., see the abstract.

 Given that this is a retrospective study, one should perhaps state that “Co-morbidities, including gastrointestinal anomaly, short bowel syndrome, Down syndrome, and cardiopulmonary function, are…” significant associated factors for severe RSV-LRTI”… rather than ‘significantly higher risk factors for severe RSV-LRTI’.

 The same comment goes for the use of the phrase ‘risk factors for severe RSV-LRTI and death’ in the introduction; however, the correct phrase is more correctly stated in the data analysis plan.

**Limitations**

  It is not completely clear in the text what proportion of the co-infections or bacterial coinfections were laboratory proven and what proportion were attending clinician diagnosed; hence the influences of these unidentified potential infections could not be resolved. If the is information is known, clarification is required in the methods section.

**Strengths**

 Despite this, the study population although not a justified sample size,  was substantial with adequate available data. The criteria for severity were well described and internationally comparable. At the end of the study the researchers have identified some associated factors for severity of RSV-related LRTI at their tertiary centre.

**Ethical considerations**

 Appropriate attention to ethical considerations.

**Strengths**

 I wish to acknowledge the huge effort the authors have invested in this study, it is generally well written and presents a significant contribution to the understanding of acute RSV-associated infection in the <5-y-age group at a single centre in Thailand. Whilst the study is overall commendable, I think it would benefit from further revision and shortening of the results section, discussion and a much shorter conclusion which directly relates to the study findings.

Is the work clearly and accurately presented and does it cite the current literature?

Yes

If applicable, is the statistical analysis and its interpretation appropriate?

Yes

Are all the source data underlying the results available to ensure full reproducibility?

Yes

Is the study design appropriate and is the work technically sound?

Yes

Are the conclusions drawn adequately supported by the results?

Partly

Are sufficient details of methods and analysis provided to allow replication by others?

Yes

Reviewer Expertise:

General and Emergency Paediatrics and Child Health

I confirm that I have read this submission and believe that I have an appropriate level of expertise to confirm that it is of an acceptable scientific standard.

# Reviewer report: Reviewer response for version 2

**DOI:** 10.5256/f1000research.165923.r279756

**Published:** 2024-05-22

**Authors:** Visal Moolasart

**Affiliations:** 1 Department of Disease Control, Bamrasnaradura Infectious Diseases Institute, Ministry of Public Health, Nonthaburi, Thailand

After reading the revised version of the manuscript titled "Factors Associated with Severe Respiratory Syncytial Virus Infection Among Hospitalized Children in Thammasat University Hospital," I find the information valuable to the understanding of RSV. I am satisfied with the manuscript.

Is the work clearly and accurately presented and does it cite the current literature?

Yes

If applicable, is the statistical analysis and its interpretation appropriate?

Yes

Are all the source data underlying the results available to ensure full reproducibility?

Yes

Is the study design appropriate and is the work technically sound?

Yes

Are the conclusions drawn adequately supported by the results?

Yes

Are sufficient details of methods and analysis provided to allow replication by others?

Yes

Reviewer Expertise:

infectious disease

I confirm that I have read this submission and believe that I have an appropriate level of expertise to confirm that it is of an acceptable scientific standard.

# Reviewer report: Reviewer response for version 1

**DOI:** 10.5256/f1000research.160633.r260846

**Published:** 2024-04-12

**Authors:** Visal Moolasart

**Affiliations:** 1 Department of Disease Control, Bamrasnaradura Infectious Diseases Institute, Ministry of Public Health, Nonthaburi, Thailand

**This manuscript delivers a compelling message and provides valuable insights into RSV infection among hospitalized children. However, there are several issues that merit further consideration.**

1. Since this study site is a tertiary care and university hospital, it's probable that most participating children are from high-risk groups with underlying conditions. Hence, it's imperative to approach the assessment of risk factors and establishment of connections with caution.
2. There is a wealth of intriguing information within this study, prompting consideration of its key message.

- **RSV infection severity varies across age categories**, spanning from 0-3 months, 12-24 months, to over 24 months. It is imperative for the author to focus on analyzing, discussing, and comparing these age-specific trends with previous study findings. Hence, exploring the stratification of RSV infection by age is an intriguing issue.
- Distinguishing symptoms exist between the severe RSV-LRTI group and the non-severe RSV-LRTI group. It is imperative for the author to discuss these differences in symptoms as they relate to **predicting the severity of the condition**.
- The impact of tertiary care on risk factors and severity underscores the significance of discussing the associated therapy **costs in this study**. It is essential to include additional information to enrich this discussion.
- The definition of the severe RSV-LRTI group serves as a foundational principle in this study. For additional clarity and credibility, it is essential to include detailed information regarding this definition along with a proper reference.

         The type of diagnosis is an intriguing topic for discussion, particularly regarding conditions such as pneumonia, bronchiolitis, and bronchitis.

 3. Please, recheck information and add information at

 -page 5 line 18-24 (below Fig1) and page 8 line 8-15 this content is not match the table. Please ensure that the data is sorted according to the table provided by the author.

 - In the table, it would be beneficial for the author to add specific symbols (such as *) to indicate significant data, along with footnotes providing explanations below the table. And add “statistically significant”.

 - in table 1, revise Sever RSV-LRTI

 - p8 line 5, revise “+”

 4. **In univariable and multivariable analysis in table 4**, There are many characteristics that significant, this reason is hospital type of study site. Preterm and hematologic disease are not significant, this is interesting issue for discussion. Please condense the discussion from page 8, line 20 to page 9, line 5 for a succinct summary.

 5. Summary: Page 10 line 7-19, Please request the author to provide a brief summary or modify the information to summarize the results of this study. Exclude any unrelated issues from the discussion.

 6. While acknowledging the limitations, this study provides valuable information. The author should consider adding details about how population selection could impact both positive and negative results, as well as strengthening the overall robustness of the study.

Is the work clearly and accurately presented and does it cite the current literature?

Yes

If applicable, is the statistical analysis and its interpretation appropriate?

Yes

Are all the source data underlying the results available to ensure full reproducibility?

Yes

Is the study design appropriate and is the work technically sound?

Yes

Are the conclusions drawn adequately supported by the results?

Yes

Are sufficient details of methods and analysis provided to allow replication by others?

Yes

Reviewer Expertise:

infectious disease

I confirm that I have read this submission and believe that I have an appropriate level of expertise to confirm that it is of an acceptable scientific standard, however I have significant reservations, as outlined above.

# Response

**Published:** 2024-05-06

**Authors:** Pornumpa Bunjoungmanee

**Affiliations:** pediatric, Thammasat University, Pathum Thani, Thailand

We are grateful to you for your time, effort, and constructive criticism. We have pasted your comments below, in bold, with responses in turn.

1. **Since this study site is a tertiary care and university hospital, it's probable that most participating children are from high-risk groups with underlying conditions. Hence, it's imperative to approach the assessment of risk factors and establishment of connections with caution.**

*               *


**Response:** *We agree that the conduct of the study in a tertiary care academic medical center may have some bias in the cohort toward children with underlying conditions* *.* *This information has been added to the eighth paragraph of the discussion* *section and to the limitations of the study.*

**2. There is a wealth of intriguing information within this study, prompting consideration of its key message**.                                                                                               

**2.1 RSV infection severity varies across age categories, spanning from** **0-3 months,** **12-24 months, to over** **24 months. It is imperative for the author to focus on analyzing, discussing, and comparing these age-specific trends with previous study findings. Hence, exploring the stratification of RSV infection by age is an intriguing issue.**

**

**Response:** *We have added a discussion on the relationship between age range and disease severity. Additionally, the age-specific trend was compared with that of a previous study, which is mentioned in the fourth paragraph of the discussion section.*

****

**2.2 Distinguishing symptoms exist between the severe RSV-LRTI group and the non-severe RSV-LRTI group. It is imperative for the author to discuss these differences in symptoms as they relate to predicting the severity of the condition.**

**

****Response:** *We have added a new paragraph to the discussion regarding the differences in symptoms between both groups, which are relevant to predicting the severity of the condition. This addition is made in the sixth and seventh paragraphs of the discussion.*

****

**2.3 The impact of tertiary care on risk factors and severity underscores the significance of discussing the associated therapy costs in this study. It is essential to include additional information to enrich this discussion.**

**

**Response:** *We have added a new paragraph to the discussion which mentions the impact of tertiary care on risk factors and severity, which are associated with therapy costs. This information has been added to the eighth paragraph of the discussion*

****

**2.4 The definition of the severe RSV-LRTI group serves as a foundational principle in this study. For additional clarity and credibility, it is essential to include detailed information regarding this definition along with a proper reference.**

**

**Response:** *The definition of severe RSV-LRTI adopted in this study diverges from the criteria outlined by the World Health Organization (WHO) and other related investigations. While the WHO guidelines predominantly hinge upon clinical symptoms and specific physiological parameters, such as SpO2 levels and chest wall indrawing, our study delineates severity based on factors associated with adverse outcomes or mortality, which impact hospital resource utilization and costs.*

*Our definition of severity, although divergent from WHO criteria, aligns with similar approaches seen in studies by Aikphaibul P, et al., Shi Tet al., and Havdal LB et al. This consistency highlights the* *reliability and applicability of our research within the broader academic conversation, providing a shared basis for assessing severe RSV-LRTI across various clinical and geographical settings.*

*Reference*

*Aikphaibul P, Theerawit T, Sophonphan J, et al.: Risk factors of severe nso respiratory syncytial virus infection in tertiary care center in Thailand. Influenza Other Respir. Viruses. 2021;15(1):64–71. 32783380 10.1111/irv.12793 PMC7767956*

*Shi T, McAllister DA, O'Brien KL, et al.: Global, regional, and national disease burden estimates of acute lower respiratory infections due to respiratory syncytial virus in young children in 2015: a systematic review and modelling study. Lancet. 2017;390(10098):946-958.*

*Shi T, Vennard S, Mahdy S, et al. Risk Factors for Poor Outcome or Death in Young Children With Respiratory Syncytial Virus-Associated Acute Lower Respiratory Tract Infection: A Systematic Review and Meta-Analysis. J Infect Dis. 2022;226(Suppl 1):S10-S16.*

*Havdal LB, Bøås H, Bekkevold T, et al. Risk factors associated with severe disease in respiratory syncytial virus infected children under 5 years of age. Front Pediatr. 2022;10:1004739. doi: 10.3389/fped.2022.1004739. eCollection 2022.*

**2.5 The type of diagnosis is an intriguing topic for discussion, particularly regarding conditions such as pneumonia, bronchiolitis, and bronchitis.**

**

**Response:** *The diagnosis of RSV-LRTI was based on medical records approved by the attending physician and ICD-10 coding from hospital discharge summaries, as outlined in the study methodology.*

****

**3. Please, recheck information and add information at**

**3.1 page** **5 line** **18-24 (below Fig** **1) and page** **8 line** **8-15 this content is not match the table. Please ensure that the data is sorted according to the table provided by the author.**

**

**Response:** *The information has been reviewed and corrected for accuracy.*

****

3.2 In the table, it would be beneficial for the author to add specific symbols (such as *) to indicate significant data, along with footnotes providing explanations below the table. And add “statistically significant”.

**

**Response:** *The symbols have been incorporated into the table with corresponding explanations provided below, indicating statistical significance.*

****

**3.3 in table** **1, revise Sever RSV-LRTI**

****Response:** *The correction has been made to replace "Sever" with "Severe" in Table 1.*

****

**3.4 p** **8 line** **5, revise “+”**

**

**Response:** *The correction has been made to replace "+" with “ +”*

                

**4. In univariable and multivariable analysis in table** **4, There are many characteristics that significant, this reason is hospital type of study site. Preterm and hematologic disease are not significant, this is interesting issue for discussion. Please condense the discussion from page** **8, line** **20 to page** **9, line** **5 for a succinct summary.**

**

**Response:** *We have expanded the discussion to include the results of preterm birth and hematologic disease in both univariable and multivariable analyses. Comments on other factors mentioned in Table 4 have been incorporated into the eighth paragraph of the discussion. The information from line 20 on page 8 to line 5 on page 10 pertains to the results section, not the discussion. Therefore, this information is retained in the manuscript.*

****

**5. Summary: Page** **10 line** **7-19, Please request the author to provide a brief summary or modify the information to summarize the results of this study. Exclude any unrelated issues from the discussion.**

**

**Response:** *We concur that this section is lengthy, and we have condensed this information in the discussion section.*

****

**6. While acknowledging the limitations, this study provides valuable information. The author should consider adding details about how population selection could impact both positive and negative results, as well as strengthening the overall robustness of the study.**

            

**Response:** We have added the strengths of this study and both the positive and negative impacts of the study population on the strengths and limitations part.
